# Effect of Sequence-Based Incorporation of Fillers, Kenaf Fiber and Graphene Nanoplate, on Polypropylene Composites via a Physicochemical Compounding Method

**DOI:** 10.3390/polym17141955

**Published:** 2025-07-17

**Authors:** Soohyung Lee, Kihyeon Ahn, Su Jung Hong, Young-Teck Kim

**Affiliations:** 1 Department of Sustainable Biomaterials, College of Natural Resources and Environment, Virginia Tech, Blacksburg, VA 24061, USAkihyeon@vt.edu (K.A.); 2 Macromolecules Innovation Institute (MII), Virginia Tech, Blacksburg, VA 24061, USA; 3 Department of Food and Nutrition, Kyung Hee University, Seoul 02447, Republic of Korea

**Keywords:** polypropylene, graphite nanoplatelets, kenaf fiber, filler addition sequence, pallet materials

## Abstract

Natural-fiber-reinforced polypropylene (PP) composites are gaining increasing interest as lightweight, sustainable alternatives for various packaging and applications. This study investigates the effect of filler addition sequence on the mechanical, morphological, thermal, and dynamic mechanical properties of PP-based composites reinforced with graphite nanoplatelets (GnP) and kenaf fiber (KF). Two filler incorporation sequences were evaluated: GnP/KF/PP (GnP initially mixed with KF before PP addition) and GnP/PP/KF (KF added after mixing GnP with PP). The GnP/KF/PP composite exhibited superior mechanical properties, with tensile strength and flexural strength increasing by up to 25% compared to the control, while GnP/PP/KF showed a 13% improvement. SEM analyses revealed that initial mixing of GnP with KF significantly improved filler dispersion and interfacial bonding, enhancing stress transfer within the composite. XRD and DSC analyses showed reduced crystallinity and lower crystallization temperatures in the addition of KF due to restricted polymer chain mobility. Thermal stability assessed by TGA indicated minimal differences between the composites regardless of filler sequence. DMA results demonstrated a significantly higher storage modulus and enhanced elastic response in the addition of KF, alongside a slight decrease in glass transition temperature (T_g_). The results emphasize the importance of optimizing filler addition sequences to enhance mechanical performance, confirming the potential of these composites in sustainable packaging and structural automotive applications.

## 1. Introduction

Pallet packaging plays a fundamental role in modern logistics and supply chains, providing a stable platform for transporting and storing a wide range of products [1]. Despite their extensive use, traditional wooden pallets face challenges such as susceptibility to moisture, fungal growth, and pest infestation, which often require additional treatments like fumigation or heat treatment [2]. Plastic pallets can be more cost-effective but often face durability issues under repeated loading and impact [3]. Consequently, there is growing demand for pallet materials that can offer improved mechanical performance, lighter weight, and enhanced environmental sustainability without excessively increasing production costs.

One emerging solution involves advanced composites that incorporate both natural fibers and carbon-based nano-reinforcements [4,5]. Among natural fibers, kenaf (*Hibiscus cannabinus*) fiber (KF) stands out due to its rapid growth rate, excellent tensile properties, and low production cost, making it highly suitable for large-scale manufacturing [6,7]. KF, when blended with polypropylene (PP), can significantly enhance tensile and flexural properties, contributing to greater structural rigidity at a moderate weight increase [8]. However, the inherently hydrophilic nature of KF can lead to weak interfacial bonding with non-polar thermoplastic matrices, thus requiring compatibilizers such as maleic anhydride grafted polypropylene (MAPP) [9,10].

To further boost the mechanical and thermal performance of PP composites, graphite nanoplatelets (GnP) have been proposed as a cost-effective alternative to more expensive carbon nanofillers like multi-walled carbon nanotubes or graphene [11]. GnP, with its two-dimensional platelet structure, provides high stiffness, good thermal conductivity, and potential improvement in wear resistance [12,13,14]. Critically, GnP is available at a market price of about $10–20 per kilogram, making it feasible for large applications such as pallets, where economics and scalability are paramount [15]. Recent studies have already shown that GnP can be dispersed successfully in a wide range of thermoplastic matrices, including polyamide-12, polyethylene and PEEK, using both melt compounding and additive-manufacturing routes. For example, 3D-printed polyamide-12 parts containing up to 10 wt% GnP exhibited an eight-order-of-magnitude jump in electrical conductivity (10^−11^ → 10^−4^ S/cm), a 34% reduction in heating time to 90 °C, and a 25% drop in friction coefficient without dimensional distortion [16]. Moreover, Kalaitzidou et al. [17] demonstrated that chemical pretreatment prior to compounding enhances the dispersion of exfoliated graphite nanoplatelets (xGnP), leading to superior mechanical performance. These results confirm the versatility of GnP as a multifunctional reinforcement for thermoplastics and motivate its combined use with natural fibers in pallet-grade composites.

Combining KF and GnP in PP-based composites has the potential to achieve synergistic reinforcement effects, where KF primarily contributes to enhanced tensile properties and impact absorption, and GnP provides improved stiffness, thermal stability, and wear resistance. Such composite systems, having cost efficiency, are particularly relevant for plastic pallets, which are routinely exposed to heavy loads, sudden impacts, and fluctuating temperature/humidity conditions [18]. Despite this promise, the dispersion quality and the sequence in which KF and GnP are incorporated into the PP matrix can significantly affect the final composite properties. Poor dispersion or unfavorable filler-matrix interactions may lead to localized stress concentrations and reduced overall performance [19,20].

Hamma et al. (2014) [21] focused solely on KF in a starch-grafted PP matrix, confirming that fiber length governs the stiffness/creep balance, while Lee et al. (2023) [22] addressed only GnP, showing that high-shear dispersion of 3 wt % GnP in PP boosts dielectric and EMI-shielding properties without considering natural fibers. These studies examined single-filler PP composites; none isolated the sequence of KF and GnP addition as an independent variable.

Because GnP readily re-aggregates once shear forces cease, and rough KF surfaces can act as physical carriers for nanosheets, we hypothesized that pre-coating KF with GnP would maximize nanosheet dispersion and fiber/matrix interfacial contact. This has not been investigated completely. The present work is therefore the first to systematically compare two compounding routes, (i) GnP → KF → PP and (ii) GnP → PP → KF, under identical formulation and processing conditions, and to link the resulting microstructure to mechanical and thermal performance metrics relevant to pallet-grade applications. This aspect is critical as the filler-adding sequence can significantly influence filler dispersion, interfacial adhesion, mechanical performance, and, ultimately, industrial processability and costs.

Therefore, this study evaluates the impact of filler addition sequences, specifically the sequence of KF and GnP incorporation, on the mechanical, morphological, thermal, and dynamic mechanical properties of PP-based composites. The findings aim to inform cost-effective manufacturing strategies, promoting the practical development of high-performance, environmentally sustainable composites for pallet packaging applications.

## 2. Materials and Methods

### 2.1. Materials

PP (Mw = 340,000; Mn = 97,000; density = 0.9 g/mL; MFI = 4 g/10 min) and MAPP were purchased from Sigma Aldrich (St. Louis, MO, USA) to serve as polymer matrix and compatibilizer, respectively, thereby enhancing interfacial adhesion between the fillers and the PP. GnP, with an average length of 10–500 nm and a thickness of 5–100 nm, was supplied by Asbury Carbons Inc. (Asbury, NJ, USA), and used as a reinforcing filler. KF (average length ≈ 2 mm; diameter 20–25 µm) was obtained from Carriage House Paper (Brooklyn, NY, USA) and used as a natural fiber reinforcement. Isopropyl alcohol (IPA) was obtained from Spectrum Chemical Mfg. Corp. (New Brunswick, NJ, USA) and used as a dispersing medium during the material preparation process.

### 2.2. Preparation of PP-Based Composites

**Figure 1:**
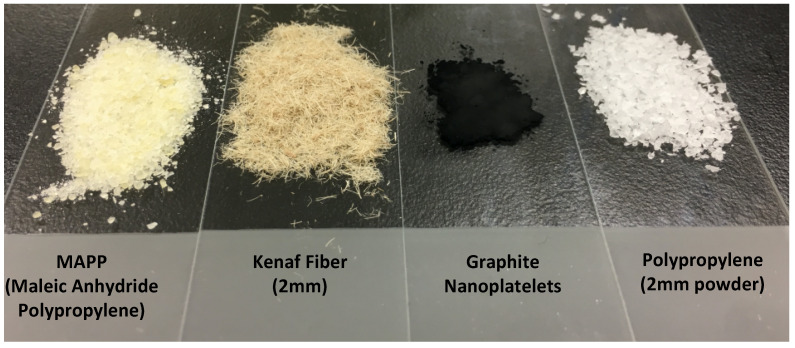
Images of polypropylene (PP), graphite nanoplatelets (G), kenaf fiber (K), and maleic anhydride polypropylene (MAPP).

PP, KF, and MAPP were first ground with a Thomas Wiley Laboratory Mill (Model 4, Thomas Co., Philadelphia, PA, USA) and sieved through a 2 mm mesh to achieve a uniform particle size. Two mixing sequences were designed to examine how the timing of KF addition influences final composite properties. The physical forms of the individual components used and their respective compositions are presented in Figure 1 and Table 1. KF was fixed at 30 wt%, which lies within the 20–30 wt% window reported to maximize tensile and flexural performance without inducing excessive brittleness or processing viscosity [8]. A lower fiber fraction (<20 wt%) provided insufficient stiffening, whereas higher levels (>35 wt%) caused mold-filling defects in our pilot trials. GnP was fixed at 0.3 wt%, the highest loading that improved stiffness without platelet agglomeration in our preliminary screening. MAPP was held at 3 wt%, the mid-point of the 2–5 wt% range reported to maximize fiber–matrix compatibilization while avoiding surplus additive. This combination provided the optimal balance between mechanical gains, dispersion quality, and material cost.

For GnP/PP/KF, GnP was dispersed in IPA and ultrasonicated for 1 h in pulse mode (on: 45 s, off: 15 s) at 40% amplitude under an ice bath to prevent overheating. PP powder was then gradually introduced under continuous ultrasonication to facilitate uniform interaction between the polymer and nanoparticles. Next, KF was added, followed by an additional ultrasonication step, and finally, MAPP was incorporated.

**Figure 2:**
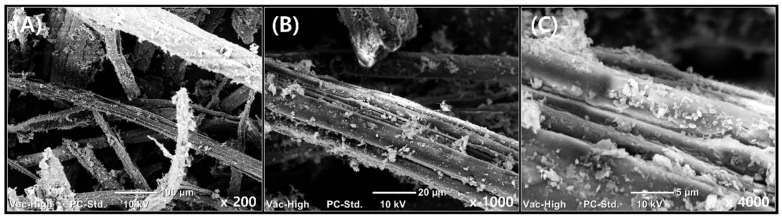
Representative microscopic images of pre-mixed GnP and kenaf at different magnifications: (**A**) ×200; (**B**) ×1000; (**C**) ×4000.

For GnP/KF/PP, GnP was dispersed in IPA and ultrasonicated under the same conditions, after which KF was gradually introduced under continuous ultrasonication. Representative images of the pre-mixed GnP and kenaf fiber prior to compounding are shown in Figure 2.

PP was then added, and MAPP was introduced last. Each suspension underwent an extra 30 min of ultrasonication to ensure thorough mixing. The mixtures were filtered, dried at 100 °C for 24 h to remove residual IPA, and compounded in a co-rotating twin-screw extruder (HAAKE MiniLab II Micro Compounder, Thermo Scientific Inc., Waltham, MA, USA) at 220 °C and 150 rpm. The extrudates were pelletized using a pelletizer (Benchtop 25 Lab Series, Bay Plastic Machinery, Bay City, MI, USA) and then molded into standard specimens using an injection molding machine (Model 150A, LNS Tech, Yucaipa, CA, USA) at a cylinder temperature (T_cylinder_) of 200 °C and a mold temperature (T_mold_) of 80 °C. A control sample (GnP/PP) without KF was prepared following the same procedure for comparison.

### 2.3. Characterization of PP-Based Composites

#### 2.3.1. Mechanical Properties

The unnotched Izod impact strength (IS) was measured using an Izod Impact Tester (CEAST 9050, Instron, Norwood, MA, USA) in accordance with ASTM D256. Tensile strength (TS) and Young’s modulus (YM) were determined using a Universal Testing Machine (MTS Systems Corporation, Eden Prairie, MN, USA) following ASTM D638, and flexural strength (FS) and flexural modulus (FM) were measured according to ASTM D790, all at room temperature.

#### 2.3.2. Dynamic Mechanical Analysis (DMA)

Dynamic mechanical analysis (DMA) was conducted using a DMA Q800 (TA Instruments, New Castle, DE, USA) to evaluate storage modulus, loss modulus, and tan delta. The dual cantilever mode was employed over a temperature range of −60 to 160 °C, at a heating rate of 5 °C/min, an oscillation frequency of 1 Hz, and a strain amplitude of 0.1%. Specimens were injection-molded into dimensions of 62 mm × 13 mm × 3 mm.

#### 2.3.3. Thermal Properties

Thermogravimetric analysis (TGA) was carried out using a TGA Q500 (TA Instruments, New Castle, DE, USA) under a nitrogen atmosphere. Samples (6–8 mg) were heated from room temperature to 600 °C at a rate of 10 °C/min. Differential scanning calorimetry (DSC) was performed using a DSC250 (TA Instruments, New Castle, DE, USA) under a nitrogen atmosphere to investigate the crystallization and melting behaviors of the composites. Each specimen was subjected to a heat–cool–heat cycle: (i) heating from −40 °C to 180 °C at 10 °C/min to erase previous thermal history, (ii) cooling back to −40 °C at the same rate, and (iii) reheating to 180 °C at 10 °C/min. The degree of crystallinity (*D*) was determined using the following Equation (1):

(1) $$D=\frac{∆H_{m}}{∆H_{0}f_{PP}}\times 100\%$$

where *D* is the degree of crystallinity, ∆*H_m_* is the melting enthalpy, ∆*H*_0_ is the melting enthalpy of 100% crystalline PP (209 J/g) [23], and *f_PP_* is the weight fraction of PP in the composite

#### 2.3.4. Morphological Analysis

Fracture surfaces of impact-tested specimens were examined using scanning electron microscopy (SEM, NeoScope JCM-5000, JEOL, Tokyo, Japan) to evaluate the morphology and interfacial adhesion between the polymer matrix and fillers. Prior to observation, all samples were sputter-coated with palladium (DESK II Coater, Denton Vacuum Inc., Moorestown, NJ, USA) to mitigate charging effects. SEM micrographs were acquired at an accelerating voltage of 15 kV.

#### 2.3.5. X-Ray Diffraction (XRD) Analysis

X-ray diffraction (XRD) analysis was performed to investigate the crystalline structure and phase composition of the samples. Measurements were conducted using a D8 Advance X-ray diffractometer (Bruker Corporation, Billerica, MA, USA) with Cu Kα radiation (λ = 1.5406 Å) operated at 40 kV and 40 mA. Diffraction patterns were collected in continuous scan mode over a 2θ range of 10–60° with a step size of 0.025° and a counting time of 0.5 s/step. The instrument was equipped with “twin/twin” optics, including a motorized primary slit (0.6 mm) and a secondary slit (5 mm), and was maintained at a cooling temperature of 15–20 °C. The resulting data in brml format were processed using PowDLL or Profex to generate spectra plotting XRD angle (2θ) versus intensity. The crystallinity index (*CrI*) of PP-based composites was calculated using the following Equation (2):

(2) $$CrI\left(\%\right)=\frac{\left(I_{200}-I_{am}\right)}{I_{200}}\times 100$$

where *I_am_* represents the peak intensity of the amorphous region, and *I*_200_ corresponds to the total intensity under the diffraction curve

### 2.4. Statistical Analysis

A nested experimental design was implemented with three independent batches, each containing five replicate samples (n = 15 total). Statistical comparisons were performed using one-way analysis of variance (ANOVA) with batch-to-batch variability as the random effect, followed by post hoc Tukey’s honestly significant difference (HSD) test for pairwise comparisons. All analyses were conducted at a 95% confidence interval (α = 0.05) using JMP Pro (v16.0.0, SAS Institute Inc., Cary, NC, USA).

## 3. Results and Discussion

### 3.1. Mechanical and Morphological Properties of PP-Based Composites

**Figure 3:**
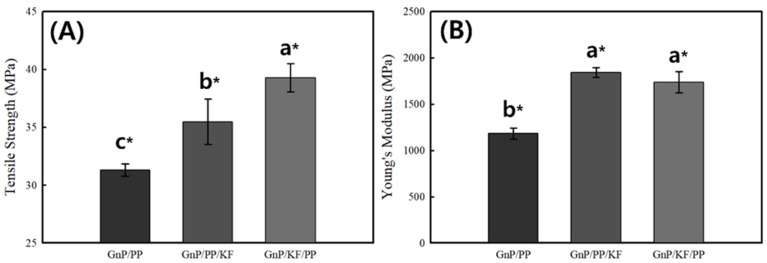
Tensile strength (**A**) and Young’s modulus (**B**) of PP-based composites as a function of GnP and KF mixing sequence. * indicates statistically significant differences between groups (*p* < 0.05), determined by Tukey’s HSD test. Data are presented as means ± standard deviations.

The TS and YM of the PP-based composites are shown in Figure 3. The control composite (GnP/PP) exhibited a TS of 31.30 ± 0.54 MPa and a YM of 1181.89 ± 48.80 MPa. Both composites reinforced with KF and GnP demonstrated significant enhancement in TS and YM. The GnP/KF/PP composite, where GnP was first mixed with KF before blending with PP, displayed the highest TS (39.27 ± 0.89 MPa), representing approximately a 25% increase over the GnP/PP. In contrast, the GnP/PP/KF composite, in which KF was added after mixing GnP with PP, showed a smaller TS improvement of about 13% (35.48 ± 1.96 MPa) than that of GnP/KF/PP. These enhancements are attributed to the optimized filler addition sequence, which promotes better dispersion of GnP and stronger KF-matrix interactions, resulting in improved load transfer and stress distribution within the matrix. SEM observations (Figure 2) further confirm that mixing GnP and KF prior to PP incorporation facilitates more uniform embedding of GnP onto the KF surface without aggregation. This result clearly shows that GnP is effectively anchored onto the KF surfaces, minimizing fiber pull-out and enhancing the interfacial bonding between fillers and the PP matrix. This structural integration enables more efficient stress transfer and significantly contributes to the observed improvements in mechanical performance. Similar observations were reported by Feng et al. [24] in kenaf/PALF fiber-reinforced PP composites, noting fiber-matrix debonding, fiber pull-out, and fiber breakage during tensile testing. Furthermore, the YM similarly reflected the influence of filler addition sequence, increasing to 1842.69 ± 43.89 MPa in GnP/PP/KF and 1736.55 ± 91.99 MPa in GnP/KF/PP. These improvements in YM are likely due to enhanced composite stiffness resulting from improved interfacial adhesion and the reinforcing effects of rigid filler interactions [25].

**Figure 4:**
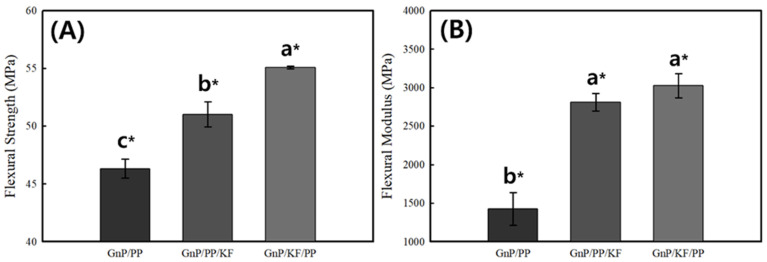
Flexural Strength (**A**) and Modulus (**B**) of PP-based composites as a function of GnP and KF mixing sequence. * indicates statistically significant differences between groups (*p* < 0.05), determined by Tukey’s HSD test. Data are presented as means ± standard deviations.

The FS and FM results of the PP-based composites are presented in Figure 4. The GnP/PP composite exhibited the lowest FS (46.32 ± 0.81 MPa) and FM (1427.23 ± 211.40 MPa). Flexural properties were significantly affected by the addition of fillers and the filler-adding sequence, leading to increased values similar to those observed in the tensile properties. The GnP/KF/PP composite showed the most pronounced improvements, achieving the highest FS (55.07 ± 0.11 MPa) and FM (3025.28 ± 156.88 MPa), while the GnP/PP/KF composite demonstrated improved flexural strength (51.01 ± 1.10 MPa) and modulus (2809.70 ± 111.71 MPa). The increase in flexural modulus of the hybrid composites can be attributed to the presence of rigid crystalline phases as well as a higher fraction of KF [26]. This indicates that the filler incorporation sequence markedly influences composite stiffness and load-bearing capacity, likely due to optimized dispersion and enhanced interfacial adhesion between the KF, GnP, and PP matrix. Particularly in plastic pallet packaging applications, increased flexural properties are highly advantageous as they directly contribute to improved load-bearing capacity and rigidity under static and dynamic loads. Higher FM translates to reduced deformation under applied loads, enhancing dimensional stability and durability of plastic pallet structures.

**Figure 5:**
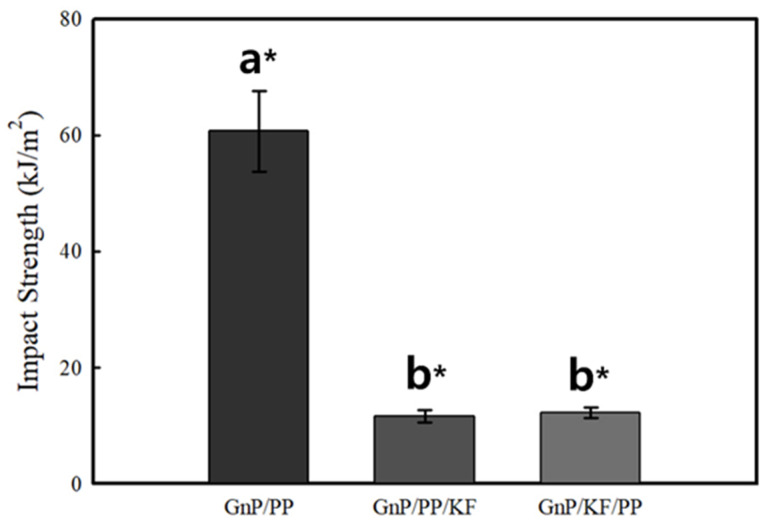
Impact Strength of PP-based composites as a function of GnP and KF mixing sequence. * indicates statistically significant differences between groups (*p* < 0.05), determined by Tukey’s HSD test. Data are presented as means ± standard deviations.

The impact test was conducted to evaluate the resistance of the composites to suddenly applied forces by measuring the impact energy absorbed prior to fracture. The IS results are presented in Figure 5. The GnP/PP composite exhibited an IS of 60.69 ± 6.92 kJ/m^2^, while the GnP/KF/PP and GnP/PP/KF composites showed IS values of 12.30 ± 0.91 kJ/m^2^ and 11.59 ± 1.07 kJ/m^2^, respectively. Although these values indicate a significant reduction compared to GnP/PP alone, both GnP/KF/PP and GnP/PP/KF composites demonstrated significantly enhanced impact resistance relative to neat PP (3.92 kJ/m^2^) reported in a previous study [27]. This substantial improvement in IS is primarily attributed to the synergistic reinforcement effects provided by GnP and KF, along with enhanced interfacial adhesion facilitated by the incorporation of MAPP. The combined action of GnP and KF effectively improved energy dissipation during impact loading, thus significantly enhancing the composite’s overall toughness and resistance to fracture compared to neat PP. According to previous studies, the fiber length is crucial in the physical and mechanical properties of the fiber-based composites. The fiber length, which was used in this study (2 mm), is relatively very short compared to the literature values. The relatively short fiber length may have further contributed to the reduced impact resistance, as long fibers are generally more effective in dissipating impact energy through fiber pull-out and crack bridging mechanisms. Similar trends have been reported in previous studies, where the incorporation of short natural fibers into thermoplastics led to a decrease in IS [28,29], whereas composites reinforced with long fibers demonstrated improved impact resistance [30]. Based on these findings, fiber length within the thermoplastic matrix plays a crucial role in determining energy absorption capability and overall mechanical performance.

**Figure 6:**
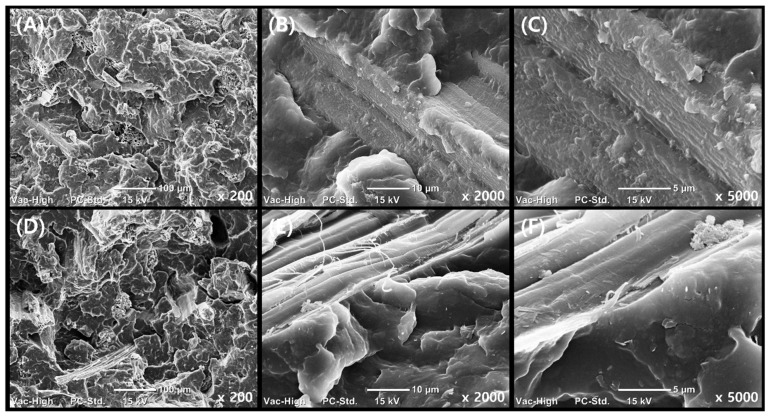
SEM images of tensile fracture surfaces of PP composites: (**A**–**C**) GnP/KF/PP; (**D**–**F**) GnP/PP/KF at ×200, ×2000, and ×5000, respectively.

SEM analysis of the tensile fracture surfaces was conducted to examine the effect of filler addition sequence on composite morphology (Figure 6).

In GnP/KF/PP composites, GnP was initially mixed with KF, resulting in uniform dispersion and effective embedding of GnP onto the KF surfaces. SEM images clearly showed minimal fiber pull-out and well-anchored GnP particles [31], indicating strong interfacial bonding and efficient stress transfer within the polymer matrix [26]. In contrast, GnP/PP/KF composites exhibited pronounced fiber pull-out and less effective embedding of GnP onto the KF surfaces, suggesting weaker fiber-matrix adhesion. These morphological findings correlate closely with mechanical test results, providing clear evidence that mixing GnP with KF prior to incorporating it into PP significantly enhances composite performance by optimizing filler dispersion and interfacial interactions.

Consequently, optimizing filler addition sequences, as demonstrated by the GnP/KF/PP composite, may yield composite materials better suited for demanding packaging applications, potentially improving plastic pallet lifespan and reliability.

### 3.2. Crystallinity of PP-Based Composites

**Figure 7:**
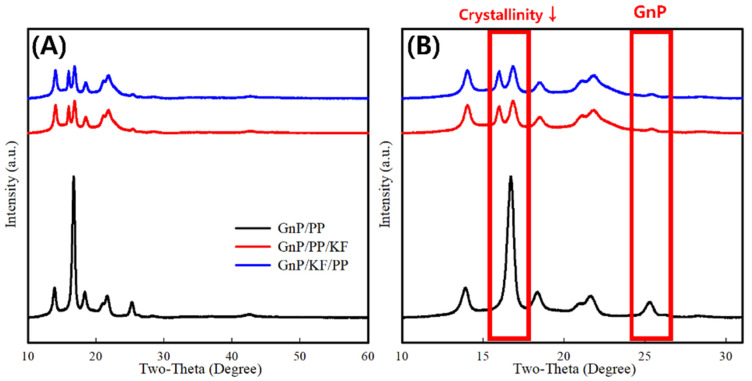
XRD patterns of PP-based composites with different filler addition sequences: (**A**) full scan (2θ = 10–60°); (**B**) magnified view of the main region (2θ = 10–30°).

XRD patterns of the PP-based composites are presented in Figure 7. A characteristic peak corresponding to GnP was observed at 2θ = 26.07° (200) across all samples [32]. PP exhibited characteristic diffraction peaks at 2θ = 14.2°, 17.0°, and 18.8°, corresponding to the (110), (040), and (130) crystal planes of the α-phase, respectively [33]. Additionally, peaks appearing between 21.1° and 22.1° represent a combination of α-phase (131 and 041) and β-phase (301) of PP [17]. Notably, the GnP/KF/PP and GnP/PP/KF composites exhibited an additional distinct peak at 2θ = 16° corresponding to the (300) plane of the β-phase, indicating that kenaf fibers act as nucleating agents promoting β-phase crystallization. Previous studies have similarly reported KF’s nucleating capability in PP composites, highlighting their role in crystallinity enhancement and secondary reinforcement mechanisms [34,35]. However, crystallinity analysis (Figure 7) showed there is no significant difference in crystallinity between GnP/KF/PP (50%) and GnP/PP/KF (51%), while showing slightly lower than that of GnP/PP (58%). This reduction suggests that, despite the nucleating effects of GnP and KF, KF may restrict polymer segmental chain mobility during crystallization, leading to decreased overall crystallinity [4,36].

### 3.3. Thermal Properties of PP-Based Composites

**Figure 8:**
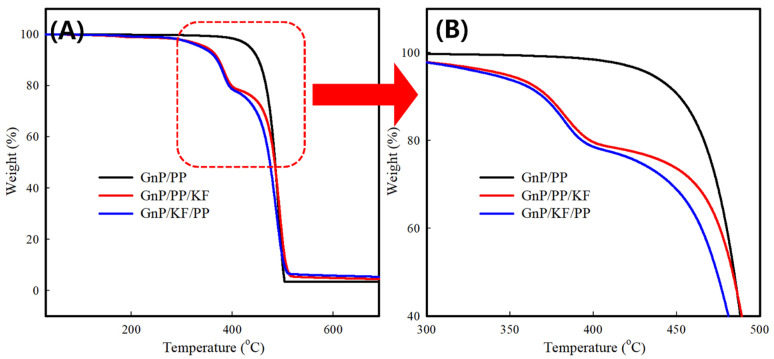
TGA curves of PP-based composites with different filler addition sequences: GnP/PP, GnP/PP/KF, and GnP/KF/PP. (**A**) Full-range TGA curves up to complete decomposition. (**B**) Magnified view of the degradation region (300–500 °C).

TGA results for PP-based composites are shown in Figure 8 and summarized in Table 2, including onset, 5%, 10% and 20% weight loss temperatures. The control GnP/PP composite displayed thermal decomposition in a single-stage process from approximately 286 °C to 498 °C. In contrast, GnP/KF/PP and GnP/PP/KF composites exhibited two distinct degradation stages: the first stage, ranging from 195 °C to 360 °C, is attributed to the scission of C–O and C–C bonds and thermal degradation of glycosidic linkages via trans-glycosylation at lower temperatures, associated primarily with cellulose and hemicellulose components of KF [37]. The second stage, occurring from approximately 395 °C to 510 °C, is primarily due to aromatization processes involving dehydration, responsible for high-temperature decomposition, and associated with PP degradation [38]. No significant differences were observed between the GnP/KF/PP and GnP/PP/KF composites during the first decomposition stage, while a slight improvement was noted in the GnP/PP/KF composite during the second stage. This may indicate that the decomposition of kenaf fiber accelerates the thermal degradation of polypropylene.

**Figure 9:**
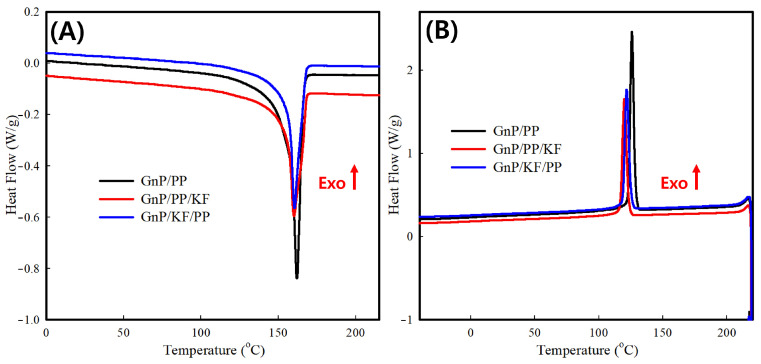
DSC thermograms of PP-based composites with different filler addition sequences: (**A**) second heating and (**B**) cooling scans. Exothermic heat flow is plotted in the upward direction.

DSC was performed to investigate the crystallization and melting behaviors of composites (Figure 9 and Table 3). Consistent with the XRD findings, DSC analysis indicated a reduction in crystallinity for GnP/KF/PP and GnP/PP/KF composites compared to the GnP/PP composite. Previous studies have reported increased crystallinity upon kenaf fiber addition [17,25,39]; however, the current results suggest that KF limited polymer chain mobility, thereby reducing crystallinity. This decrease is attributed to the hindered segmental alignment of polymer chains during crystallization due to the presence of KF.

### 3.4. Viscoelastic Properties of PP-Based Composites

**Figure 10:**
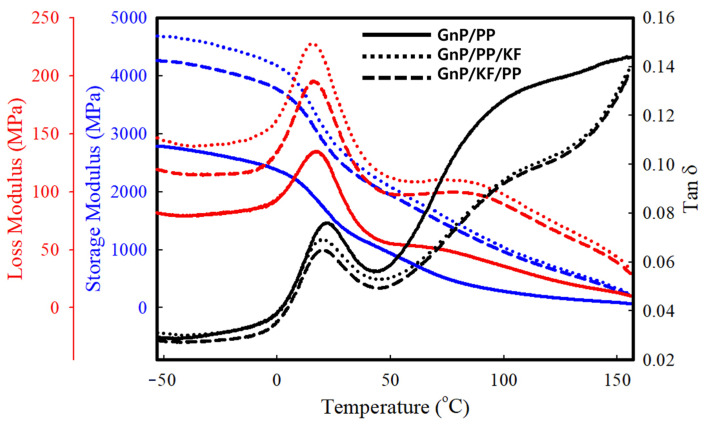
Storage modulus, loss modulus, and tan δ of PP-based composites with different filler addition sequences (GnP/PP, GnP/PP/KF, and GnP/KF/PP) as a function of temperature.

Figure 10 presents the storage modulus and tan delta curves of PP-based composites as a function of temperature ranging from −60 °C to 160 °C. The glass transition temperature (Tg), determined from the tan delta peak, did not differ between GnP/PP/KF (20.1 °C) and GnP/KF/PP (20.4 °C); both were slightly lower than that of GnP/PP (22.3 °C). This reduction aligns with the observed decrease in crystallinity, as lower crystallinity typically correlates with a reduced Tg in semi-crystalline polymers, reflecting transitions occurring over a broader temperature range [17]. The storage modulus, indicative of a material’s ability to store elastic energy, significantly increased with the incorporation of KF, indicating enhanced elastic behavior under loading. This improvement can be attributed to kenaf fibers restricting the mobility of polypropylene chains, thereby increasing overall composite stiffness [40]. Similar trends of increased storage modulus upon natural fiber addition have been reported previously, suggesting effective matrix reinforcement by natural fibers [41,42]. Furthermore, the storage modulus of GnP/KF/PP was slightly higher than that of GnP/PP/KF, indicating that the filler addition sequence also affects viscoelastic behavior. This difference may be attributed to enhanced interfacial bonding and more homogeneous filler dispersion when GnP is pre-mixed with KF, as evidenced by SEM observations. For all composites, a continuous decline in storage modulus was observed above Tg, reflecting the elastic-dominated behavior of PP composites in the extended rubbery regime (Tm—Tg) at higher temperatures. Additionally, the difference in modulus behavior above 80 °C between KF-reinforced and control suggests that fiber-matrix interactions and crystalline structure changes become more prominent at elevated temperatures. The control sample, GnP/PP, exhibited a significantly lower loss modulus (E′′) compared to both GnP/PP/KF and GnP/KF/PP, as shown in Figure 10. This increase is attributed to the addition of kenaf fiber, which enhances the material’s ability to dissipate energy as heat during deformation, resulting from increased internal friction within the composite due to the presence of the kenaf fibers. A similar phenomenon has been well reported in the literature reviewed by Haris et al. [43]. This temperature-dependent divergence is consistent with previous reports that associate DMA thermograms with crystalline phase evolution at higher temperatures [44].

## 4. Conclusions

In this study, the effects of incorporating GnP and KF into PP composites, along with varying the filler addition sequence, were comprehensively evaluated. The composite prepared by first mixing GnP with KF (GnP/KF/PP) exhibited superior mechanical performance, including higher TS, FS, and FM compared to the composite with KF added after GnP and PP mixing (GnP/PP/KF). SEM analyses confirmed that initial mixing of GnP with KF enhanced filler dispersion and significantly improved interfacial interactions between fillers and the PP matrix, resulting in more effective stress transfer. XRD results consistently indicated reduced crystallinity in composites containing KF, attributed to restricted polymer chain mobility caused by KF’s presence. TGA revealed no significant differences in thermal stability between the two filler addition sequences, with KF influencing thermal degradation stages primarily due to its inherent characteristics. DMA analyses further confirmed increased stiffness and improved elastic response of the addition of KF. These results indicate that optimizing the filler-adding sequence is crucial for enhancing mechanical properties in PP-based composite fabrication. The findings have significant implications for developing sustainable and environmentally friendly composite materials for structural applications, such as pallet packaging and automotive components, by leveraging the renewable and biodegradable advantages of natural fibers. Looking forward, two practical issues remain. First, the long-term durability of the GnP-pre-coated fiber network under cyclic humidity and UV exposure has yet to be verified, as does modulus retention after multiple recycling passes. Second, the scalability of the GnP pre-coating step must be demonstrated at the pilot-plant level to confirm economic viability. Future work will, therefore, quantify property retention after UV-B exposure, hygrothermal cycling, and repeated mechanical reprocessing, establishing the durability and recyclability required for pallet-grade applications.

## Figures and Tables

**Table 1:** Composition of the PP-based composites.

Samples	PP (wt%)	MAPP (wt%)	KF (wt%)	GnP (wt%)
GnP/PP	96.7	3.0	0.0	0.3
GnP/PP/KF	66.7	3.0	30.0	0.3
GnP/KF/PP	66.7	3.0	30.0	0.3

**Table 2:** Thermal Degradation Temperatures (T_d_) of PP-based composites with different filler addition sequences, based on TGA.

Sample	T_d5%_ (°C)	T_d10%_ (°C)	T_d20%_ (°C)
GnP/PP	435	452	467
GnP/PP/KF	348	373	398
GnP/KF/PP	339	370	393

**Table 3:** DSC data of PP-based composites with different filler addition sequences, based on TGA.

Sample	Crystallization Point			Melting Point			Crystallinity (%)
	Onset Temp. (°C)	Peak Temp. (°C)	Enthalpy (J/g)	Onset Temp. (°C)	Peak Temp. (°C)	Enthalpy (J/g)	
GnP/PP	128 ± 0	126 ± 0	97 ± 0	158 ± 0	162 ± 0	95 ± 3	46 ± 1
GnP/PP/KF	123 ± 0	120 ± 0	70 ± 1	156 ± 0	160 ± 0	66 ± 1	32 ± 1
GnP/KF/PP	125 ± 0	122 ± 0	70 ± 2	156 ± 0	161 ± 0	67 ± 3	33 ± 2

## Data Availability

Data will be made available on request.
